# COVID-19 pandemic: choosing a loose-fitting PAPR for better protection? Add an N95 FFR

**DOI:** 10.1186/s42077-021-00156-4

**Published:** 2021-05-03

**Authors:** Omprakash Srinivasan, Vinodhadevi Vijayakumar, Arimanickam Ganesamoorthi

**Affiliations:** 1 Pediatric Cardiac Anesthesiologist, Pediatric Cardiac Care, MIOT Hospitals, Chennai, Tamil Nadu India; 2 Department of Anesthesiology and Critical care, Meenakshi Hospital, Thanjavur, 613005 India

**Keywords:** COVID-19 pandemic, Powered air-purifying respirator (PAPR), N95 Filtering Facepiece Respirator (FFR)

Respected Editor,

Powered air-purifying respirator (PAPR) is a respirator that uses a blower to force air through filter cartridges or canisters into the breathing zone of the wearer through a tight-fitting half or full facepiece or a loose-fitting facepiece, hood, or helmet (Centre for Disease Control 2020).

Loose-fitting National Institute for Occupational Safety and Health (NIOSH)-approved PAPRs have several advantages over tight-fitting non-powered respirators (Centre for Disease Control 2020). They are:

1. PAPR systems have Assigned Protection Factor (APF) of at least 25 (may up to 1000) while N95 Filtering Facepiece Respirator (FFR) have an APF of 10 (Centre for Disease Control 2020). PAPRs are recommended for use in extremely aerosolizing procedures of the airway, lung, sinus, oropharynx, and skull base surgery (American Society of Anesthesiologists 2020).
2. Fit test is not needed (Centre for Disease Control 2020).
3. Splash protection for the face and eyes (Centre for Disease Control 2020).
4. Less taxing physiologically from a breathing resistance perspective than other respirators (Centre for Disease Control 2020).

In a loose-fitting PAPR, the exhaled air of the wearer is not passed through a filter. This exhaled air could contaminate the sterile surgical field and more importantly it could become source of infection to others.

Centre for Disease Control (CDC) has raised concerns about the use of PAPRs and respirators with exhalation valves for operating room (OR) use, because the exhaled air would not be filtered (American Society of Anesthesiologists 2020). Regular surgical mask may be worn under a PAPR or over the expiratory valve of the facemask respirator to afford standard protection from clinician’s exhaled air in OR (American Society of Anesthesiologists 2020).

Evidence suggests that SARS-CoV-2 transmission occurs from symptomatic and asymptomatic healthcare worker (HCW) to patients and other healthcare workers (Lucey et al. 2020). A HCW wearing a loose-fitting PAPR may become a source of infection during pre-symptomatic/asymptomatic phase or after returning back to work post-recovery. When the healthcare worker returns to work after recovering from the illness, the exact criteria that determine which HCWs will shed replication-competent virus for longer periods are not known (Centre for Disease Control 2020). On the other end, the wearer of a loose-fitting PAPR could become susceptible to infection during potential ‘No mask exposures’.

1. Immediately before donning the loose-fitting PAPR.
2. Until complete doffing and wearing a N95 FFR.

Surgical face masks protect against SARS-CoV-2 droplet transmission but not against aerosolized particles (American Society of Anesthesiologists 2020). When a loose-fitting PAPR is chosen for additional protection, it should not be used alone and it is prudent to wear a N95 FFR in addition (Fig. 1). If the HCW failed fit test for tight-fit respirators, it is a good practice to wear at least a surgical mask under a PAPR.

**Fig. 1:**
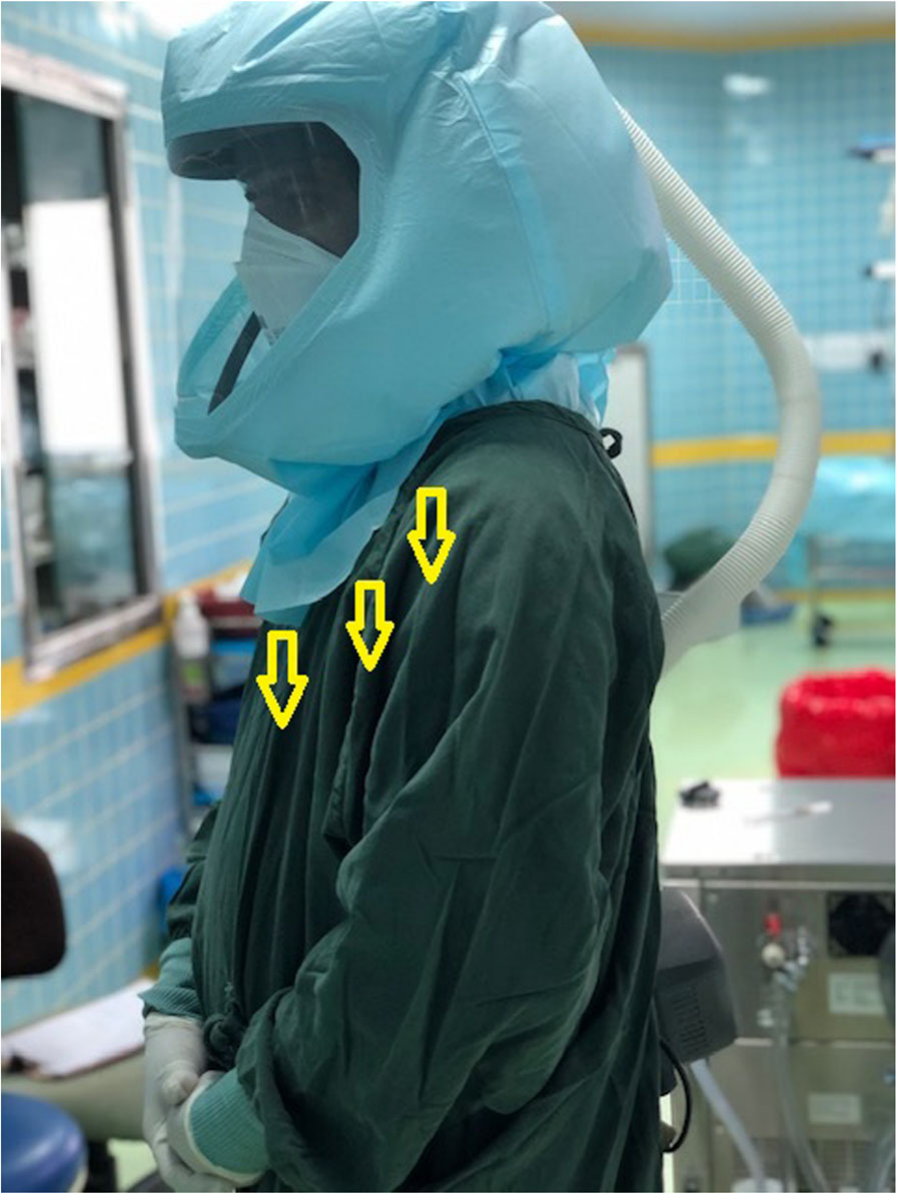
An Anesthesiologist wearing a N95 FFR along with loose-fitting PAPR. The arrows indicate the direction of the exhaled air escaping under loose-fitting hood but filtered through the N95 FFR

Wearing N95 FFR under a PAPR protects the wearer during donning and doffing and during mechanical or battery failure (Roberge et al. 2008), provides a sterile surgical field, and prevents infection spread to others. With increased production and availability of N95 FFR, this strategy of combining N95 FFR with loose-fitting PAPR is possible.

## Data Availability

Not applicable

## Abbreviations

COVID-19: Coronavirus disease—2019
SARS-CoV-2: Severe acute respiratory syndrome—coronavirus 2
PAPR: Powered air-purifying respirator
NIOSH: National Institute for Occupational Safety and Health
APF: Assigned Protection Factor
N95 FFR: N95 Filtering Facepiece Respirator
CDC: Centre for Disease Control
OR: Operating room
HCW: Healthcare worker

## References

[CR1] American Society of Anesthesiologists (2020) REVISED: The use of personal protective equipment by anesthesia professionals during the COVID-19 pandemic (June 3, 2020) Joint Position Statement n.d. https://www.asahq.org/about-asa/newsroom/news-releases/2020/06/revised-the-use-of-personal-protective-equipment-by-anesthesia-professionals-during-the-covid-19-pandemic. Accessed 12 Dec 2020

[CR2] Centre for Disease Control (2020) Considerations for optimizing the supply of powered air-purifying respirators (PAPRs) for healthcare practitioners (HCP) https://www.Cdc.Gov/Coronavirus/2019-Ncov/Hcp/Ppe-Strategy/Powered-Air-Purifying-Respirators-Strategy.Html. Accessed 12 Dec 2020

[CR3] Centre for Disease Control (2020) Interim operational considerations for public health management of healthcare workers exposed to or with suspected or confirmed COVID-19: non-U.S. n.d. Healthcare settings. https://www.cdc.gov/coronavirus/2019-ncov/hcp/non-us-settings/public-health-management-hcw-exposed.html. Accessed 12 Dec 2020

[CR4] Lucey M, Macori G, Mullane N, Sutton-Fitzpatrick U, Gonzalez G, Coughlan S et al (2020) Whole-genome sequencing to track SARS-CoV-2 transmission in nosocomial outbreaks. Clin Infect Dis:ciaa1433. 10.1093/cid/ciaa1433 10.1093/cid/ciaa1433 PMC7543366 32954414

[CR5] Roberge MR, Vojtko MR, Roberge RJ, Vojtko RJ, Landsittel DP (2008). Wearing an N95 respirator concurrently with a powered air-purifying respirator: effect on protection factor. Respir Care.

